# Supplementary material for: Genetic screen of the yeast environmental stress response dynamics uncovers distinct regulatory phases
Source: Mol Syst Biol. 2019 Aug 27;15(8):e8939. doi: 10.15252/msb.20198939 (PMC6711295; doi:10.15252/msb.20198939)
Supplement: Supplementary file 1 — Expanded View Figures PDF [file MSB-15-e8939-s001.pdf]

Expanded View Figures

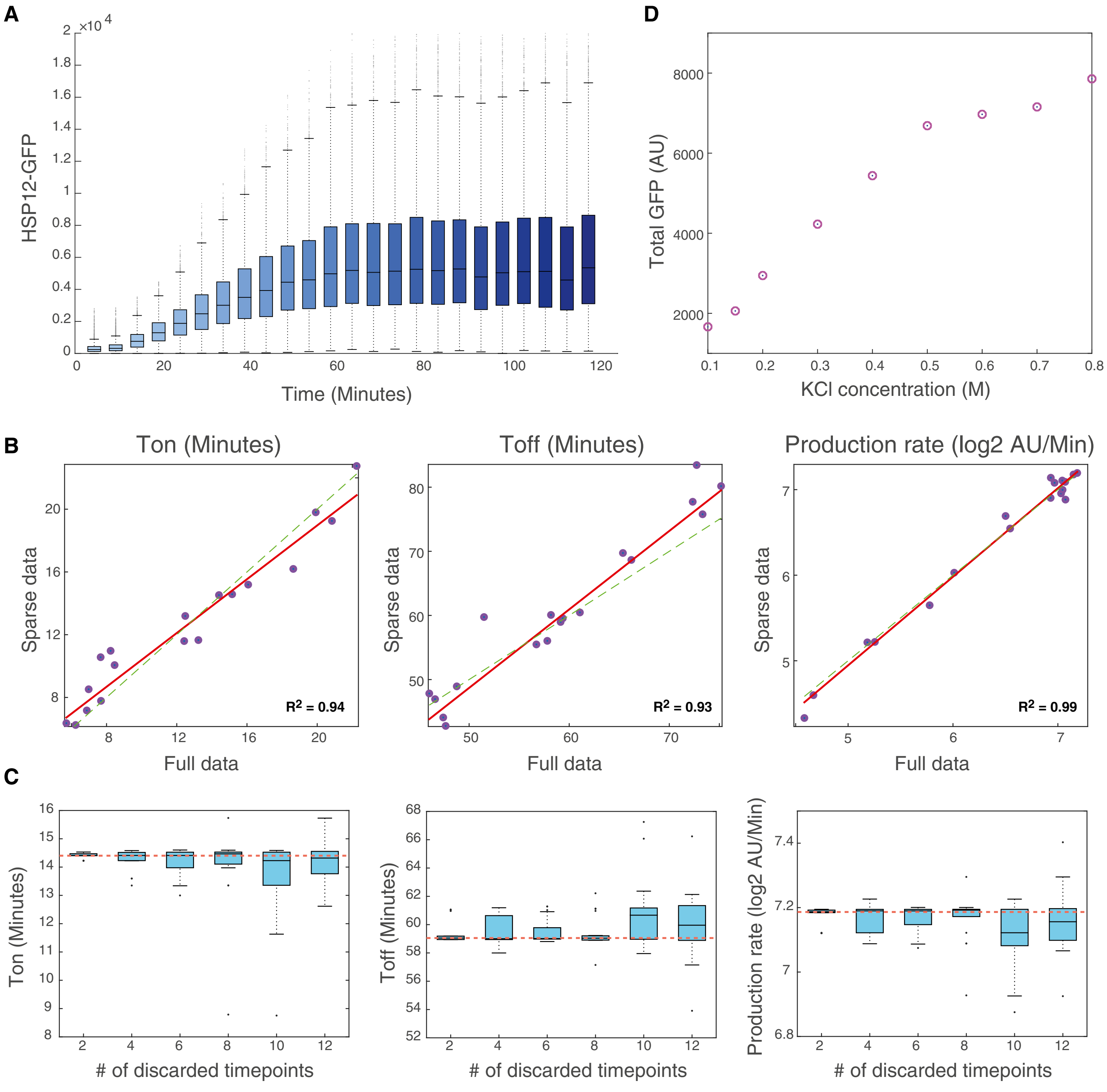

Figure EV1.

Figure EV1. A parametric view of dynamic transcriptional response.

A Response of HSP12-GFP to 0.4 M KCl on WT background. The *x*-axis show the time after introduction of KCl and the *y*-axis show the GFP levels (AU). Boxplots show the distribution of values in the populations measured at each time point (n > 2,500 cells per time point). Middle line 50% percentile, box 25–75% percentiles, whiskers show ± 1.5 inter-quantile range (IQR, 75%–25% width) from box limits. Dots show outliers from the whisker range.

B Effect of sparser time resolution on parameter estimation. For each of the time series in Fig 1A, we re-estimated the parameters using only 7 equally spaced time points (out of 24). *X*-axis—estimate using full data. *Y*-axis—estimate using sparse data. Each point represents an estimate for a specific time series.

C Robustness of parameter estimates to different levels of sparsity. Using the time series of 0.4 M KCl (Fig 1A), we randomly removed a number (*x*-axis) of points from the data and re-estimated the parameters. The boxplots showing the estimates of the parameters in 20 independent repeats of the procedure (boxes as in A). The dashed red line shows the parameter estimate from full data. Note the narrow range of estimated values for each parameter.

D Total GFP produced (*y*-axis) as a function of KCl concentration (*x*-axis) from the experiment shown in Fig 1A. The total GFP parameter was estimated as the maximum of the curve.

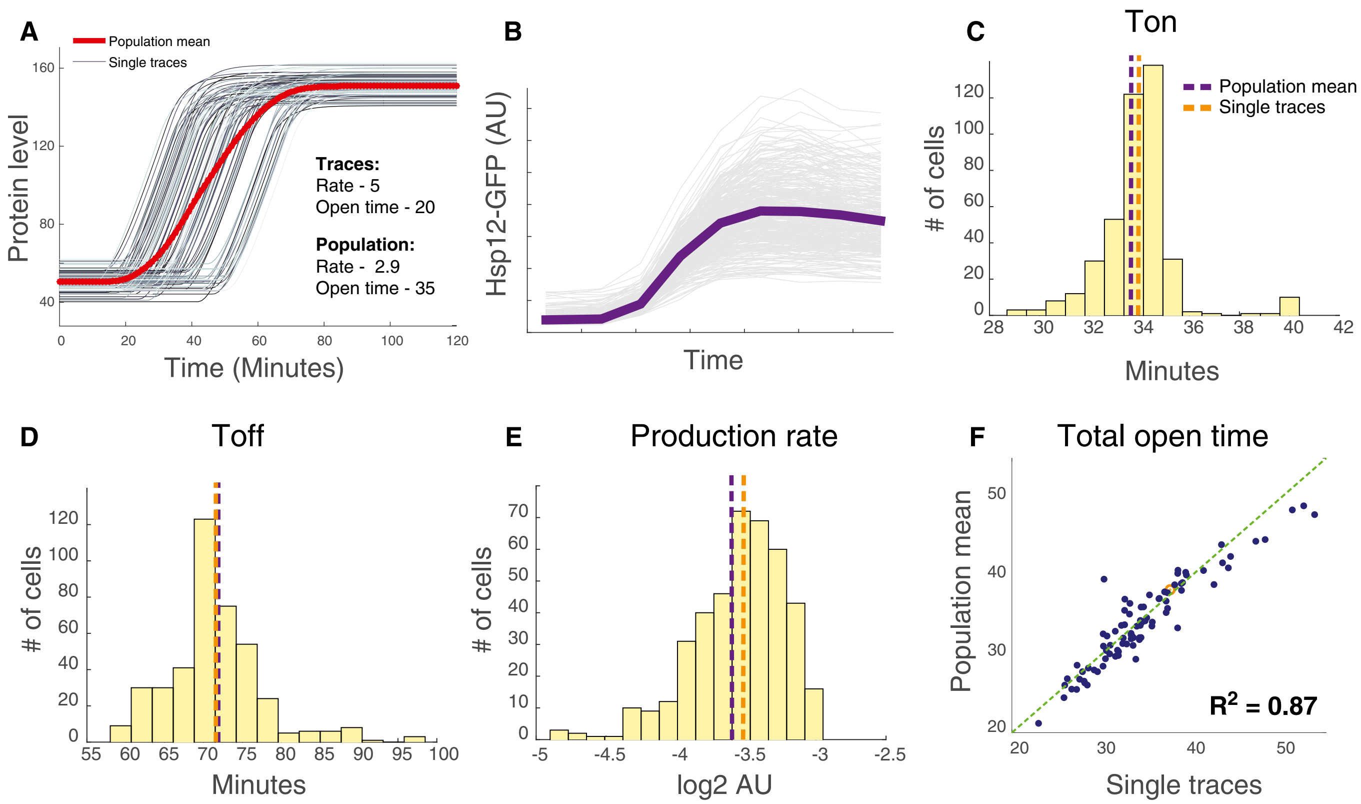

Figure EV2. Partially decoupled regulation of transcriptional dynamic parameters inferred from protein and RNA measurements.

A Illustration of a potential artifact of a parameter estimation from the population mean. Shown is the simulation of GFP induction in single cells with exactly the same production rate and total open time, but different onset times. The red line shows the mean of the population at each time point. The parameters estimated from the population curve are different from the parameters of the individual curve (inset).

B Population mean of the individual traces shown in Fig 2H.

C–E Histogram of the T_{on} parameter (C), the T_{off} parameter (D), and the production rate parameter (E) derived from individual traces (Fig 2H). The orange dashed line marks the mean of the histogram, and the purple line marks the value of the parameter estimated from the population mean (B).

F Scatter plot showing the total open time estimated from single traces versus the total open time estimated from the population average. Each point represents a different yeast strain.

 © 2019 The Authors

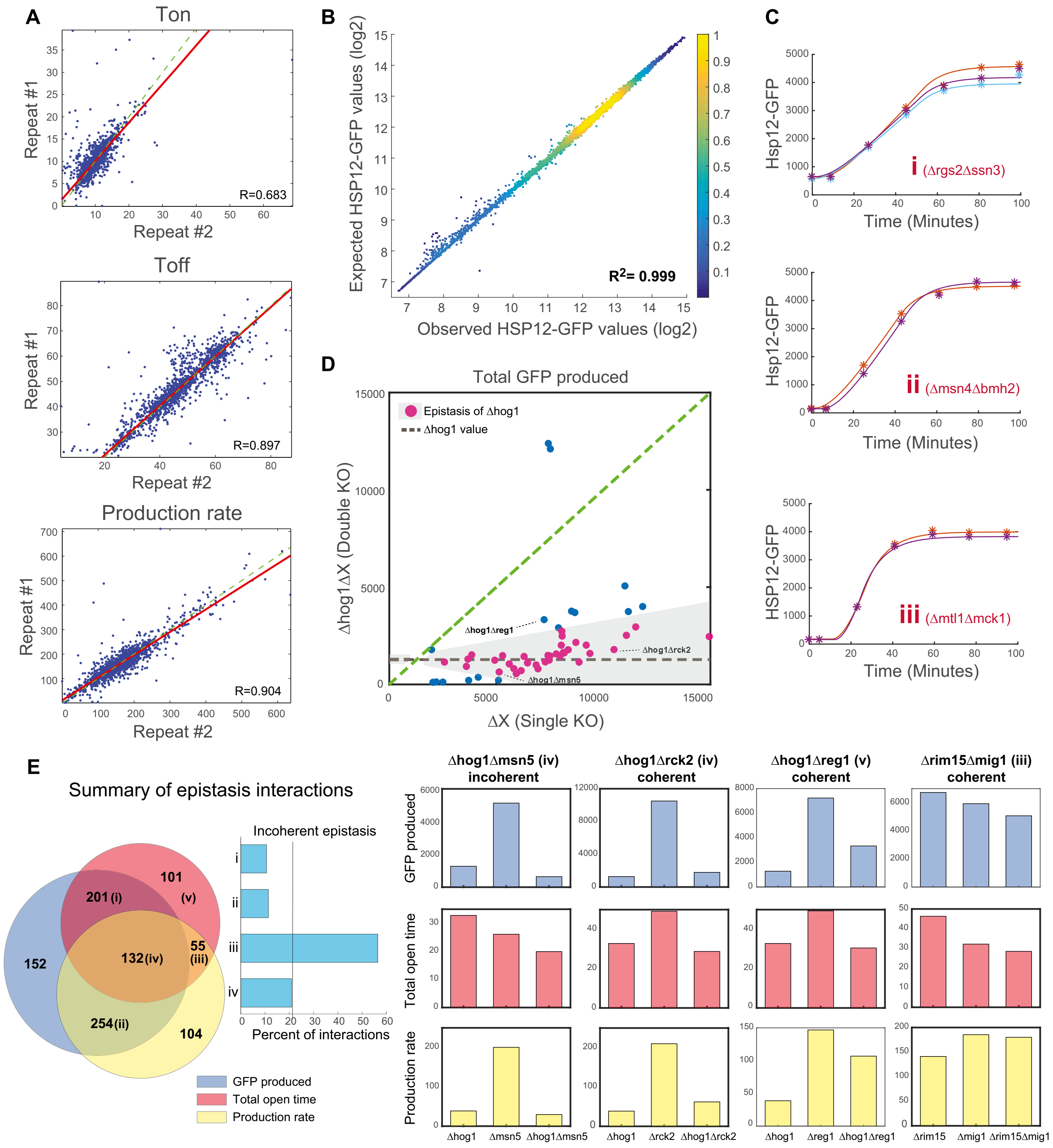
A
Ton
Repeat #1
Repeat #2
R=0.683
Toff
R=0.897
Production rate
R=0.904
B
Expected HSP12-GFP values (log2)
Observed HSP12-GFP values (log2)
R2= 0.999
C
Hsp12-GFP
Time (Minutes)
i (Δrgs2Δssn3)
ii (Δmsn4Δbmh2)
HSP12-GFP
iii (Δmtl1Δmck1)
D
Total GFP produced
Epistasis of Δhog1
Δhog1 value
Δhog1ΔX (Double KO)
ΔX (Single KO)
Δhog1Δreg1
Δhog1Δrck2
Δhog1Δmsn5
E
Summary of epistasis interactions
152
201 (i)
101 (v)
132 (iv)
55 (iii)
254 (ii)
104
Incoherent epistasis
Percent of interactions
GFP produced
Total open time
Production rate
Δhog1Δmsn5 (iv) incoherent
Δhog1Δrck2 (iv) coherent
Δhog1Δreg1 (v) coherent
Δrim15Δmig1 (iii) coherent
Δhog1 Δmsn5 Δhog1Δmsn5
Δhog1 Δrck2 Δhog1Δrck2
Δhog1 Δreg1 Δhog1Δreg1
Δrim15 Δmig1 Δrim15Δmig1

Figure EV3.

◀ **Figure EV3. Double-mutant screen for regulators of dynamic parameters.**

A Reproducibility of the parameter estimation from the double-mutant screen. Each point is a double-mutant strain. The *xy*-axis shows the results from two independent biological repeats.

B For each data point collected in the screen (strain X time point X repeat), we plot the expected value according to the estimated parameters (*y*-axis) versus the actual value (*x*-axis).

C Biological repeats of individual strains with the same total expression but different dynamics. As shown in Fig 3C.

D Illustration of the interactions of Δhog1 in the total GFP parameter. Shown the total GFP of the single mutant (*x*-axis) versus the total GFP of the double mutant with Δhog1 (*y*-axis). The gray line marks the Hog1 single-mutant levels. Points close to the gray line are ones where the value of the double mutant is close to the value of Δhog1 and defined as epistasis of Δhog1 (pink dots).

E Venn diagram comparing the number of epistatic pairs detected in various phenotypes (left). For all pairs of mutants with 2 or more types of epistatic interactions, we plotted the fraction of pairs with incoherent interactions—where Δx is epistatic in one parameter and Δy in the other (center). Examples of epistatic interactions in different regions of the venn diagram. For each pair, the values of the single mutants and the double mutant are shown for each parameter. The color of the bars matches the colors in the venn diagram. Epistatic cases are marked with bold border (right).

 © 2019 The Authors

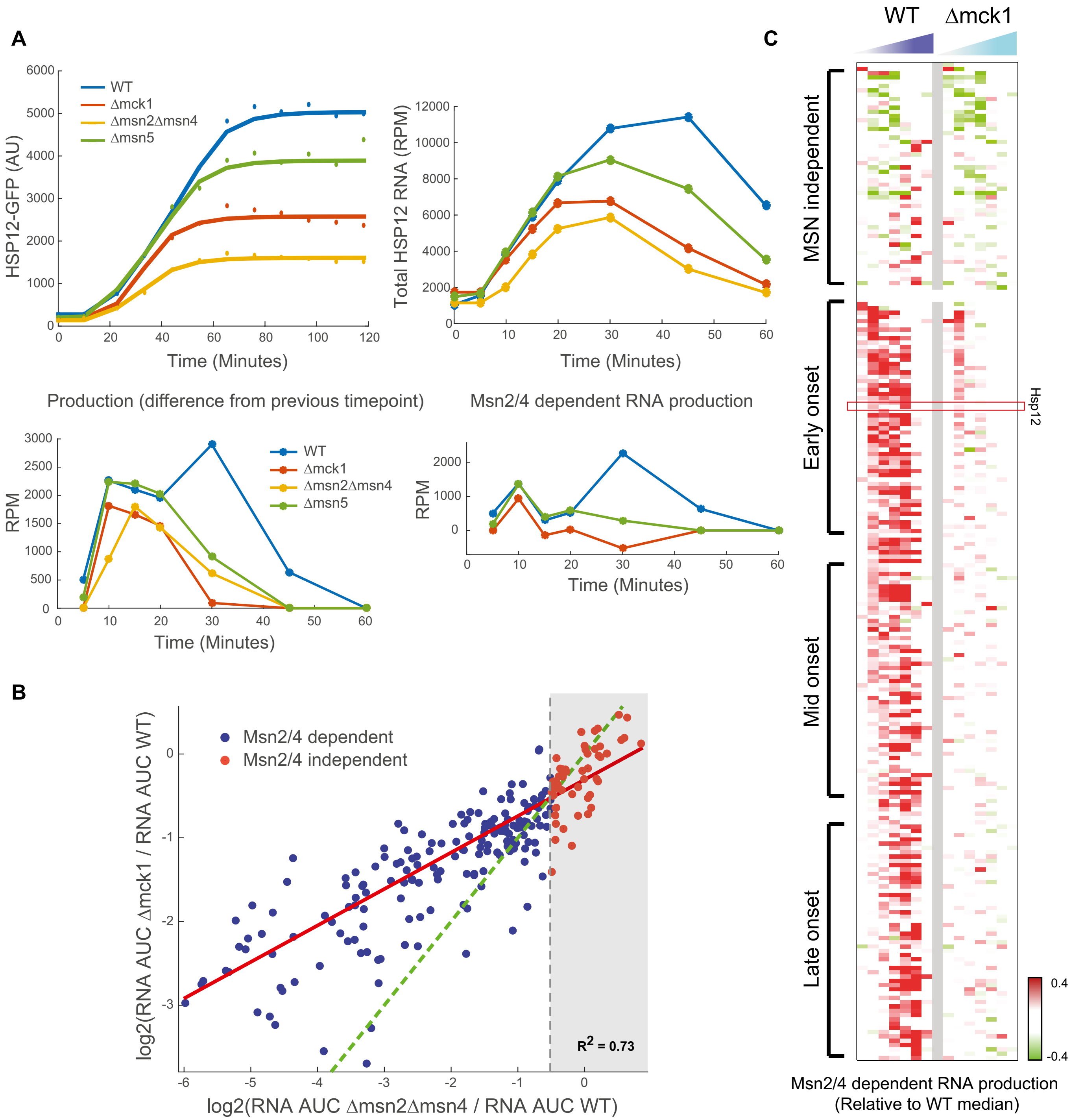

Figure EV4. MCK1 is necessary for secondary wave of Msn2/4 activity.

A Estimation of Msn2/4-dependent production of *HSP12* gene. Top left panel shows HSP12-GFP response in the different strains. Top right panel shows the normalized counts (PPM) of *HSP12* mRNA molecules in response to osmotic stress in these strains (as in Fig 4C). Bottom left panel shows the change in RNA from previous time point as a proxy for RNA production during that period. Bottom right panel is the difference of each curve in the middle panel from Δmsn2Δmsn4 production. This value captures excess production beyond Msn2/4-independent production and defined as Msn2/4-dependent production.

B The effect of Δmck1 on stress genes induction partially phenocopies the effect of Δmsn2Δmsn4. For each stress-induced gene, we estimated the change in observed RNA in Δmsn2Δmsn4 (*x*-axis) versus Δmck1 (*y*-axis) when compared to WT. Genes that had little or no effect (ratio to WT > −0.5) of Δmsn2Δmsn4 are marked in red and defined as Msn2/4 independent.

C Heatmap showing Msn2/4-dependent production of stress-responsive genes in WT and Δmck1 (in the same order as in Fig 4E).

© 2019 The Authors

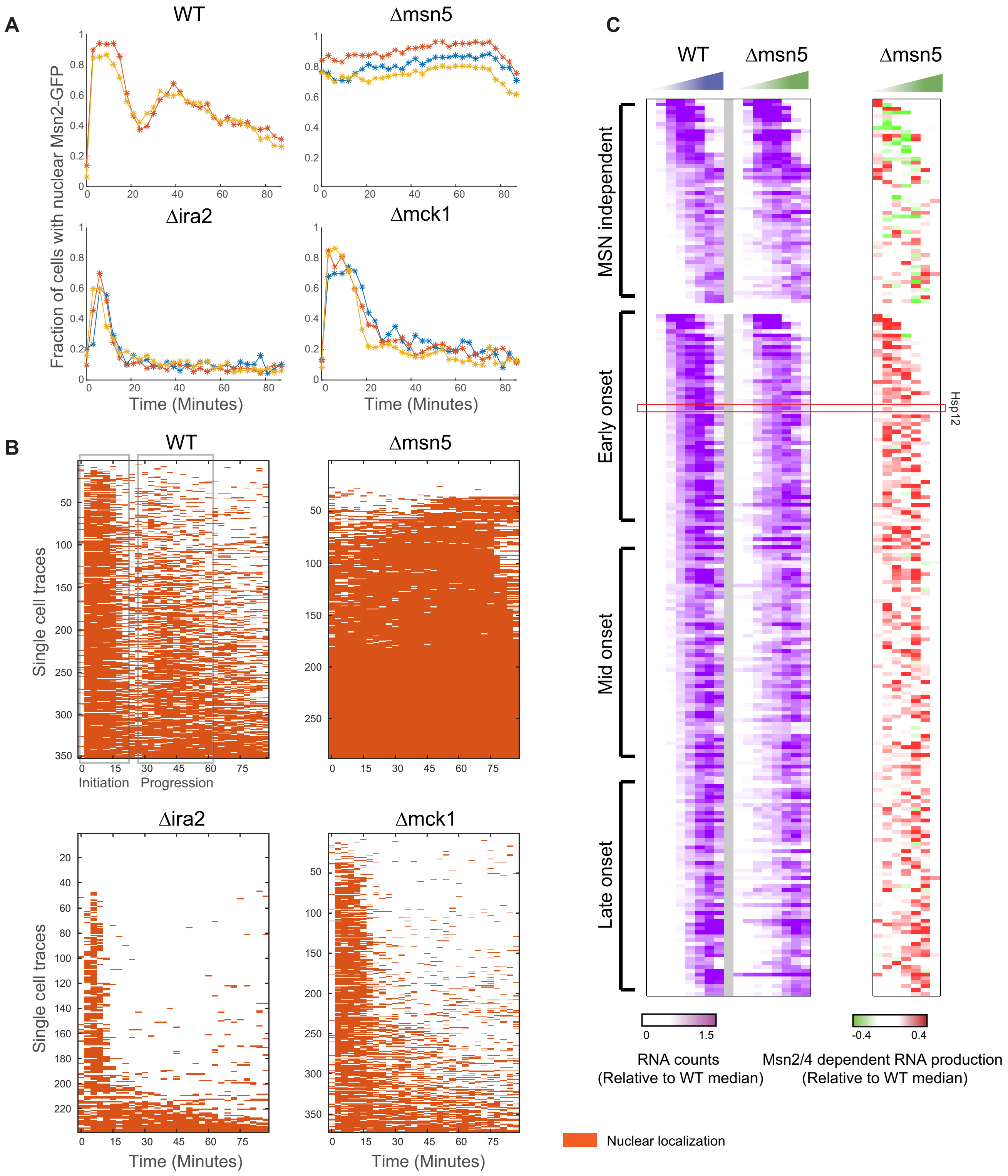

Figure EV5. Two regulatory phases in Msn2 response.

A Biological repeats of Msn2-GFP localization experiment (see Fig 5A).

B Localization pattern in individual cell traces. Each row shows the in (orange)/out (white) nuclear localization pattern of an individual cell as a function of time (*x*-axis) for the 4 strains.

C Same as Figs 4E and EV4C, for Δmsn5 strain.

 © 2019 The Authors
